# Health-related quality of life predicted subsequent health care resource utilization in patients with active cancer

**DOI:** 10.1007/s11136-018-2085-z

**Published:** 2018-12-12

**Authors:** Regina Rendas-Baum, Denise D’Alessio, Jakob Bue Bjorner

**Affiliations:** 1 Optum Insights, Inc., Johnston, RI USA; 2 Novartis Pharmaceuticals Corp., East Hanover, NJ USA

**Keywords:** Cancer, Quality of life, Health care utilization, Medical expenditures

## Abstract

**Purpose:**

The objective of this study was to estimate the association between SF-12v2® Health Survey (SF-12v2) scores and subsequent health care resource utilization (HCRU) among patients with cancer.

**Methods:**

We analyzed 18+ year participants in the Medical Expenditure Panel Survey, diagnosed with active cancer or malignancy (*n* = 647). HCRU was measured by total medical expenditures (MEs) and number of medical events (EVs) in the 6 months following the SF-12v2 assessment. The effect of SF-12v2 scores (physical (PCS) and mental (MCS) component summary scores and the SF-6D health-utility score) on HCRU was estimated using generalized linear models. Estimates were obtained for the entire sample and for the four cancer groups present in the sample: breast, prostate, skin, and lung.

**Results:**

For PCS and MCS, a one-point better score was associated with 2% lower MEs (*P* < 0.001) and 2.5% lower MEs (*P* = 0.015), respectively. A 0.05-point better SF-6D score was associated with 7% lower MEs (*P* = 0.003). PCS and SF-6D were more strongly associated with MEs for prostate cancer patients (*P* = 0.009 and *P* = 0.003) and PCS was more strongly associated with MEs for skin cancer patients (*P* = 0.019), compared to other cancer groups. A 1-point better PCS predicted 1% lower EVs, while a 0.05 better SF-6D score predicted 4% lower EVs.

**Conclusions:**

The significant associations between SF-12v2 scores from oncology patients and subsequent HCRU can guide interpretations of SF-12v2 scores in evaluation of therapies and in health policy decisions.

## Introduction

The Agency for Healthcare Research and Quality estimated that in 2014 the cost of oncology health care in the United States (US) was $87 billion [1]. Various factors have contributed to an increase in total medical expenditures (MEs) [2] bringing about a pressing need to conduct comparative evaluations of medical interventions. Such value-based appraisals of therapies are rapidly becoming an integral part of the body of evidence informing reimbursement decisions. While the cost of therapies can be objectively measured, assessments of many health outcomes (such as symptom severity, functional impacts, and quality of life) are based on subjective evaluations that reflect patient’s experiences. The patient-reported outcomes (PROs) used to collect these data are often not well understood by the various stakeholders involved in medical decision-making [3, 4]. Hence, there is a clear need to translate scores obtained from PRO measures to other metrics, such as MEs or number of hospitalizations, which are better understood by stakeholders. Additionally, linking PRO scores to real-world outcomes has the potential to further their use in clinical settings [5–7], creating opportunities for collaborative decision-making between patients and health care providers [8, 9].

Overall, the impact of specific score differences in generic health-related quality of life (HRQoL) on oncology health care resource utilization (HCRU) outcomes is not well studied nor quantified. Previous research has studied the link between PRO scores and HCRU in patients with cancer, showing that measures of symptom severity and health status are associated with outcomes related to HCRU, such as emergency room visits [10–13], hospitalizations [11, 13, 14], outpatient visits [11–13], post-operative complications [11, 15], MEs [12, 16], and use of medications [13]. Only one study [11] has calculated the impact of specific score differences on the use of health care. In that study it was estimated that each 5-point increase on the functional well-being score of the Functional Assessment of Cancer Therapy—General Population (FACT-GP) was associated with a 27% decrease in the odds of post-operative morbidity, which included emergency room and outpatient visits.

The objective of this study was to estimate a quantitative link between PRO scores and HCRU over a period of approximately 6 months following assessment of HRQoL among patients with cancer. The PRO scores included the physical and mental component summary scores of the SF-12v2® health survey (SF-12v2), a widely used generic measure of HRQoL, and the SF-6D, a utility score that can be derived from the SF-12v2 [17]. Data stemmed from the Medical Expenditure Panel Survey (MEPS), a large-scale observational study conducted in the US.

## Methods

### Data source

The MEPS is a panel-based, nationally representative survey study of approximately 15,000 households, their medical providers, and employers across the US [18]. As part of MEPS, each panel is interviewed five times at intervals of approximately 6 months, spanning two calendar years. Household interviews consist of a series of questions on specific topics (i.e., demographic characteristics, health conditions, satisfaction with care). After completion of the household interview, MEPS also contacts a sample of medical providers to obtain supplemental information, such as dates of visits, use of medical care services, charges, and sources of payments. Expenditure estimates in MEPS are based on self-reported medical care events and information from medical providers obtained through a follow-back survey. MEPS data are directly available from the following website: https://meps.ahrq.gov/mepsweb/.

### Study sample

The analyses used MEPS data from adult (18 years old or older) participants who completed the SF-12v2 and reported being diagnosed with cancer or a malignancy that was not in remission. MEPS participants were identified as an active oncology patient if they answered “yes” to the question “Have you ever been diagnosed as having cancer or a malignancy of any kind?” and “No” to the question: “Is <*cancer or malignancy*> in remission, that is, is the <*cancer or malignancy*> under control?” Data from these patients were extracted from the MEPS consolidated yearly files of 2008 (panels 12 and 13), 2010 (panels 14 and 15), and 2012 (panels 16 and 17), and the medical event files for the same MEPS panels. Each yearly file provides a single SF-12v2 assessment by subjects from two different panels, which was linked to the expenditures and medical events reported in the medical event files for the reference period subsequent to the SF-12v2 assessment.

Cancer is included in the priority conditions section within the MEPS interview. Priority conditions were selected because of their relatively high prevalence and well-developed standards for appropriate clinical care. Condition data were collected at the person-by-round level (indicating if the person was ever diagnosed with the condition) and at the condition level (as expenditures and events associated with the condition). The type of cancer or malignancy was noted verbatim by the MEPS interviewer and recoded into standard cancer malignancy types indicating the type of organ affected (e.g., breast, brain, colon). Based on the cancer malignancy codes included in MEPS, a cancer type variable was created for cancers reported by 30 or more patients. Cancer diagnoses with a lower response frequency count were coded in MEPS as “other.”

### Sociodemographic

Participant information was included as covariates to control for the differential effects of sociodemographic variables. They include age, gender, marital status (not married, married), education (less than a high school degree, high school degree, some college or associate degree, college degree, graduate education), employment status (not employed during round, employed during round), insurance status (insured any time, not insured), and number of medical conditions. As part of MEPS, medical conditions were identified by spontaneous self-report, after being probed for particular diagnoses during the priority conditions section, or any medical event or disability days linked to a specific condition.

### Health-related quality of life

**Fig. 1:**
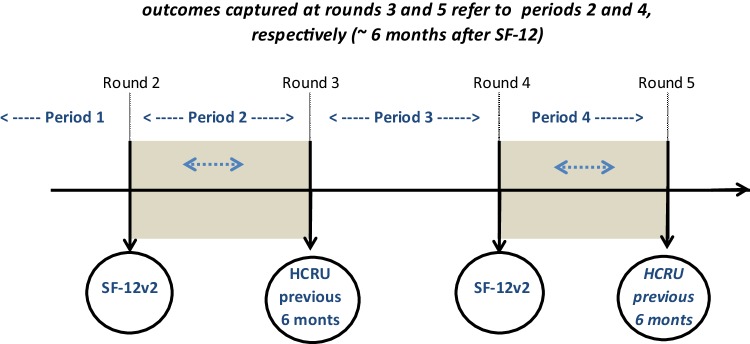
Data collection and reference periods of key outcome variables used in the current study

The SF-12v2 is a 12-item survey for measuring functional health and well-being from patients’ point of view. It consists of eight domains: physical functioning, role limitations due to physical problems, bodily pain, general health, vitality, social functioning, role of limitations due to emotional problems, and mental health. The two component summary scores (PCS and MCS) are derived from the eight domain scores and are *T* scores with a mean of 50 and standard deviation of 10 in the US general population [19]. Higher PCS and MCS scores represent better health. The SF-6D is a health-utility measure that can be scored from the SF-12v2 [17] on a metric anchored by 1 (perfect health) and 0 (death). The current scoring used utility weights from Brazier et al. [17]. The SF-12v2 survey has been used and validated to assess health status of patients with cancer [20–25]. The SF-12v2 was captured in rounds two and four of MEPS, which occur approximately 1 year apart (Fig. 1). As previously described, each consolidated yearly file includes data from two panels. Thus, for the earlier panel of each yearly file the SF-12v2 assessment occurred in round four, while for the latter panel it occurred in round two (e.g., for 2008, the SF-12v2 was captured in round two for panel 12 and in round two for panel 13).

### Health care resource utilization (HCRU)

In MEPS, data for medical events that occurred within a calendar year are provided in seven separate data files, according to the type of care: prescribed medicines, hospital inpatient stays, emergency room visits, outpatient visits, office-based provider visits, home health, and other medical expenses. The round in which the event occurred is indicated for each event, along with the total expenditures associated with the event. In the current study, these data were extracted from the medical event files associated with panels 12 through 17, as described above, and totals calculated for the reference period between the round of the SF-12v2 assessment and the subsequent round (e.g., when the SF-12v2 was captured in round four, the HCRU reference period is the time that occurred between interview rounds four and five, please see Fig. 1). For each person, total MEs were calculated as the sum of out-of-pocket or insurance payments pertaining to all seven sources described above, while utilization frequency was calculated as the sum of inpatient, emergency room, outpatient, and office-based provider events.

### Statistical analyses

For each of the two HCRU outcomes, MEs and utilization frequency, models were developed separately for PCS, MCS, and SF-6D. In addition to the SF-12v2 score, all initial models (full base models) used age, gender, marital status, education, employment status, insurance status, and number of comorbid conditions as covariates. For each of the models, diagnostics tests were conducted to determine the best form of each of the four generalized linear models (GLMs) used to estimate the effect of SF-12v2 score differences on MEs and number of medical events. The GLM is a generalization of linear regression that allows for response variables that have non-normal error distributions and has the general form $g(E[Y])=X\beta$. Under this approach, the dependent variable, *Y*, is assumed to be generated from a particular distribution that approximates its mean and variance relationship. The function, *g*, links the mean of *Y*, E[*Y*], to the linear predictor, $X\beta$ (with $X$ the vector of independent variables and $\beta$ the vector of regression coefficients). The modified Park test [26] was used to identify the distribution family that most closely described the mean–variance relationship of the data. The test identifies the best fitting distribution by comparing the coefficient from a residuals-based regression to the following values: 0 indicates Normal; 1 indicates Poisson; 2 indicates Gamma; 3 indicates Inverse Gaussian. Three additional tests were conducted that have been recommended [27] to identify the best link for the GLM: (1) Pearson correlation, (2) Pregibon link, (3) modified Hosmer and Lemeshow.

For MEs, the modified Park test indicated the Gamma to be the best distribution family (PCS coefficient = 1.94; MCS coefficient = 2.07; SF-6D coefficient = 1.94) with the log providing an appropriate link to both GLMs linking each of the SF-12v2 scores to MEs. For utilization, frequency diagnostic tests indicated that neither the Poisson nor the Normal distribution could be ruled out as adequate distributions for the GLM (PCS coefficient = 0.29; MCS coefficient = 0.34; SF-6D coefficient = 0.46) and that the log link was appropriate for the model. Additional tests for overdispersion indicated that the scale parameter in the Poisson model was significantly different from zero (*χ*^2^ = 1978.4, *P* < 0.001) and that this generalization of the Poisson model further improved model fit. Therefore, the usual Poisson model was adjusted to allow for overdispersion (with the Pearson’s chi-squared as the adjustment to standard errors).

After confirming the best distribution and link function, model refinement was carried out using at least one of the following procedures: (1) excluding, in turn, coefficients that showed no significant association with the HCRU outcome (age and gender were included in a final model regardless of statistical significance); (2) collapsing factor variables of redundant categories (i.e., those categories represented by model parameters with an associated *P* > 0.05); (3) adding model parameters representing the interaction between SF-12v2 PCS (or MCS or SF-6D) with age and gender to determine whether the effect varied according to either of these variables. After a parsimonious model was obtained (final model), a variable representing cancer types (with 30 or more patients) and its interaction with the HRQoL score was added to the model to evaluate possible differences in the effect of HRQoL on the outcome by type of malignancy. All analyses were conducted with Stata software (Version 13).

## Results

### Sociodemographic and clinical characteristics

The MEPS sample of patients with active cancer (*n* = 647) was predominantly female (57%) and the mean age was 62 years (range 18–85 years; Table 1). Forty-five percent had at least a high school degree and about 20% had a college degree or higher. Slightly over half of the sample was married (51%) and about two-thirds (67%) were not working during the time of the MEPS interview. The SF-12v2 was completed by 87% of the sample of patients with active cancer. Overall, participants reported more physical (mean = 38.2, standard deviation (SD) = 13.7) than mental health impairment (mean = 46.9, SD = 11.8). The SF-6D score had a mean of 0.665 and an SD of 0.166. The four most frequently occurring cancer types in the MEPS sample were prostate, breast, skin (combined non-melanoma, unknown type, and melanoma), and lung. Nearly all participants (95%) had one single active malignancy. A relatively small percentage of the sample had 2 (4.6%) or 3 (0.6%) malignancies. Patients with lung cancer reported the worst impairment in scores (PCS: mean = 29.8, SD = 11.7; MCS: mean = 42.5, SD = 11.3, SF-6D: mean = 0.582, SD = 0.146), among all patients with cancer. They also had, on average, more comorbid conditions (mean = 6, SD = 5.7). Patients with prostate cancer reported the highest functioning (PCS: mean = 42.5, SD = 12.5; MCS: mean = 51.2, SD = 9.8, SF-6D: mean = 0.742, SD = 0.148) and fewer comorbid conditions (mean = 4.6. SD = 3.9) than other cancer groups.

**Table 1:** Sociodemographic characteristics of analysis sample

	All cancers (*N* = 647)		Other (*N* = 315)		Prostate (*N* = 78)		Breast (*N* = 70)		Skin (*N* = 141)		Lung^a^ (*N* = 43)	
Gender, *N* (%) males^c^	281	(43.4)	123	(39.0)	78	(100.0)	0	(0)	60	(42.6)	20	(46.5)
Age, mean (SD)	62.2	(16.2)	58.7	(17.3)	69.4	(11.0)	62.2	(15.0)	64.6	(15.3)	67.4	(13.4)
Education, *N* (%)^b,c^												
< HS	159	(24.6)	87	(27.6)	14	(17.9)	16	(22.9)	28	(19.9)	14	(32.6)
HS	291	(45.0)	138	(43.8)	34	(43.6)	37	(52.9)	61	(43.3)	21	(48.8)
Some college/assoc. degree	63	(9.7)	35	(11.1)	6	(7.7)	2	(2.9)	16	(11.3)	4	(9.3)
College	74	(11.4)	31	(9.8)	11	(14.1)	10	(14.3)	20	(14.2)	2	(4.7)
> College	54	(8.3)	19	(6.0)	12	(15.4)	5	(7.1)	16	(11.3)	2	(4.7)
Missing	6	(0.9)	5	(1.6)	1	(1.3)	0	(0)	0	(0)	0	(0)
Marital status, *N* (%)^c^												
Not married	305	(47.1)	169	(53.7)	23	(29.5)	38	(54.3)	52	(36.9)	23	(53.5)
Married	332	(51.3)	141	(44.8)	53	(67.9)	31	(44.3)	87	(61.7)	20	(46.5)
Missing	10	(1.5)	5	(1.6)	2	(2.6)	1	(1.4)	2	(1.4)	0	(0)
Employment status, *N* (%)^b^												
Not employed during round	434	(67.1)	200	(63.5)	53	(67.9)	51	(72.9)	90	(63.8)	29	(67.4)
Employed during round	198	(30.6)	92	(29.2)	19	(24.4)	17	(24.3)	49	(34.8)	6	(14.0)
Missing	15	(2.3)	23	(7.3)	6	(7.7)	2	(2.9)	2	(1.4)	8	(18.6)
Number cancers not in remission, *N* (%)												
One	613	(94.7)	306	(97.1)	73	(93.6)	68	(97.1)	136	(96.5)	30	(69.8)
Two	30	(4.6)	8	(2.5)	5	(6.4)	2	(2.9)	5	(3.5)	10	(23.3)
Three	4	(0.6)	1	(0.3)	0	(0)	0	(0)	0	(0)	3	(7.0)
Number of conditions, mean (SD)^b^	5.0	(4.3)	5.1	(4.5)	4.6	(3.9)	5.3	(3.5)	4.5	(4.1)	6.0	(5.7)
PCS, mean (SD)	38.2	(13.7)	37.2	(13.4)	42.5	(12.5)	36.4	(12.7)	41.4	(14.5)	29.8	(11.7)
MCS, mean (SD)	46.9	(11.8)	45.6	(11.9)	51.2	(9.8)	46.7	(11.5)	48.9	(12.0)	42.5	(11.3)
SF-6D, mean (SD)	0.67	(0.17)	0.64	(0.160)	0.74	(0.15)	0.64	(0.15)	0.70	(0.17)	0.58	(0.15)

*MCS* mental component summary, *PCS* physical component summary, *SD* standard deviation, *HS* high school

^a^One subject counted in this group also had breast cancer

^b^Statistically significantly associated with PCS; for categorical variables, ANOVA-based *F* test indicated a *P* value < 0.05; for continuous variables Spearman correlation coefficient was both above 0.3 and had a corresponding *P* value < 0.05

^c^Statistically significantly associated with MCS; for categorical variables, ANOVA-based *F* test indicated a *P* value < 0.05; for continuous variables Spearman correlation coefficient was both above 0.3 and had a corresponding *P* value < 0.05

### Medical expenditures and utilization frequency

The utilization frequency and 6-month MEs are summarized in Table 2 by cancer type. Patients with lung cancer had the highest total mean MEs ($15,974; median = $19,421; maximum = $69,289), followed by patients with “other,” breast, prostate, and skin cancer ($3943; median = $1323; maximum = $71,532). Patients with lung cancer also had the highest mean expenditures for emergency visits, outpatient visits, office-based visits, home health, prescriptions, and other medical costs. Cost of hospital inpatient care was the greatest contributor to total expenditures with mean values that ranged between $11,411 (breast cancer) and $20,189 (“other”). The mean utilization frequency was also highest among patients with lung cancer, with 12 events, and twice as high as the average number of events among patients with prostate and skin cancer. The maximum number of medical events was 68, for the entire sample, and lowest among patients with breast cancer [31].

**Table 2:** Mean (SD) medical expenditure and utilization frequency in the 6-month period after HRQL assessment, by cancer type

	All cancers	Other	Prostate	Breast	Skin	Lung
Total expenditures	$8053	$9733	$4552	$8477	$3943	$15,974
	($15,700)	($18,914)	($8440)	($11,152)	($9437)	($19,421)
Emergency room (ER)	$1165	$1447	$338	$970	$364	$1495
	($1627)	($1869)	($275)	($747)	($471)	($1609)
Hospital inpatient care (IP)	$18,006	$20,189	$13,040	$11,411	$17,294	$18,870
	($24,428)	($29,393)	($11,097)	($6734)	($21,152)	($8153)
Outpatient visits (OP)	$4873	$5988	$3109	$4198	$1710	$11,350
	($11,343)	($13,733)	($5328)	($5864)	($1917)	($21,270)
Office-based visits (OB)	$2106	$2195	$992	$3266	$965	$5568
	($5035)	($4201)	($1406)	($7531)	($1564)	($11,614)
Other medical (OM)	$790	$727	$573	$786	$521	$1583
	($1464)	($1255)	($526)	($1008)	($663)	($2918)
Home health (HH)	$7470	$7257	$2572	$4346	$7052	$16,185
	($10,506)	($8232)	($3600)	($5419)	($6199)	($22,621)
Prescription medicines (RX)	$1610	$1820	$1106	$2195	$978	$1971
	($3722)	($4396)	($1477)	($5341)	($1191)	($2496)
Total number of OB, OP, IP, and ER events	8 (9)	8 (11)	6 (6)	9 (8)	6 (6)	12 (12)

*SD* standard deviation

### Association between SF-12v2 scores and medical expenditures

Table 3 shows parameters for final model linking PCS to MEs, which in addition to PCS, included age, gender, education, marital status, cancer type, and an interaction term between cancer type and PCS. To obtain a more parsimonious model, parameters for the lung and breast cancer groups were constrained to zero given that neither of these groups significantly differed from the “other” cancers group in terms of MEs. Similarly, the interaction term indicated that the association between PCS and MEs was significantly different for patients with skin and prostate cancer when compared to patients with the “other” cancer category (*P* ≤ 0.05), but not for the lung and breast cancer groups (*P* > 0.05). PCS differences had the strongest impact among patients with prostate cancer, followed by patients with skin cancer, and smaller for the remaining three types of cancer. After controlling for age, gender, education, and marital status, a one-point better (higher) PCS score was associated with 6% (relative risk [RR] = 1.060; 95% confidence interval [CI] [1.026, 1.094]) lower MEs in prostate cancer, 4% (RR = 1.045; 95% CI [1.021, 1.070]) lower MEs in skin cancer, and 1% (RR = 1.012; 95% CI [0.997, 1.026]) lower MEs in the remaining cancer groups (“other,” breast, lung). Age (*P* = 0.011) and marital status (*P* = 0.024) were significantly associated with MEs.

**Table 3:** Model parameters for final model linking PCS and medical expenditures, after inclusion of cancer types

Parameter	Exp (*B*)^a^	SE (*B*)	*Z*	*P* value	95% CI
Constant	6006.27	2634.95	19.83	< 0.001	2542.04–14191.44
PCS^c^	0.99	0.01	− 1.61	0.107	0.97–1.00
Cancer type—other (reference)					
Prostate	2.78	2.29	1.24	0.217	0.55–14.01
Breast	1.00	(restricted)^b^			
Skin	1.24	0.77	0.35	0.727	0.37–4.22
Lung	1.00	(restricted)^b^			
Cancer type by PCS (interaction)					
Prostate	**0.95**	**0.02**	**− 2.63**	**0.009**	**0.92–0.99**
Breast	1.00	(restricted)^b^			
Skin	**0.97**	**0.01**	**− 2.35**	**0.019**	**0.94–0.99**
Lung	1.00	(restricted)^b^			
Age	**2.11**	**0.62**	**2.55**	**0.011**	**1.19–3.73**
Gender (male)	1.23	0.21	1.26	0.208	0.89–1.71
Education—< HS (reference)					
HS	1.45	0.28	1.94	0.052	1.00–2.10
College/assoc. dgr	1.41	0.36	1.35	0.178	0.85–2.34
College or higher	1.01	0.20	0.06	0.952	0.68–1.50
Married	**0.72**	**0.10**	**− 2.26**	**0.024**	**0.54–0.96**

Values shown in bold are statistically significant (*P* < 0.05) model parameters

*PCS* physical component summary, *SE* standard error, HS high school, *CI* confidence interval

^a^For the GLM with log link, the exponentiated coefficient, Exp (*B*), of a continuous predictor is the multiplicative effect on the outcome of a one unit change in the predictor when the other continuous predictors are equal to 0 and the categorical predictors are at their reference category. For categorical predictors, the exponentiated model coefficient for category J represents the ratio of the mean of the category J and the mean of the reference category

^b^Inclusion of the cancer group variable in the model indicated that MEs of breast and lung cancer types did not differ significantly from the MEs of subjects in the “other” category. Therefore, those parameters were constrained to zero

^c^When PCS was excluded from the model, Akaike information criteria increased 5% from 10,029.0 to 10,592.8 and Bayesian information criteria increased 5% from 10,079.8 to 10,631.4

The parameters of the final model linking MCS to MEs are displayed in Table 4. Age was significantly associated with MEs (*P* = 0.001). Overall, a one-point higher (better) MCS score was associated with approximately 2% (RR = 1.019; 95% CI [1.003, 1.034]) lower MEs and no meaningful/significant differences across cancer types were identified in the association between MCS and MEs.

**Table 4:** Model parameters for final model linking MCS and medical expenditures, after inclusion of cancer types

Parameter	Exp (*B*)^a^	SE (*B*)	*Z*	*P* value	95% CI
Constant	6006.27	2634.95	19.83	< 0.001	2542.04–14191.44
MCS^c^	**0.98**	**0.01**	**− 2.44**	**0.015**	**0.97–1.00**
Cancer type—“other” (reference)					
Prostate	**0.34**	**0.09**	**− 4.07**	**0.000**	**0.20–0.57**
Breast	1.00	(restricted)^b^			
Skin	**0.34**	**0.08**	**− 4.71**	**0.000**	**0.22–0.53**
Lung	1.00	(restricted)^b^			
Age	**3.18**	**1.10**	**3.35**	**0.001**	**1.62–6.25**
Gender (male)	1.23	0.24	1.08	0.279	0.85–1.79

Values shown in bold are statistically significant (*P* < 0.05) model parameters

*MCS* mental component summary, *SE* standard error, *CI* confidence interval

^a^For the GLM with log link, the exponentiated coefficient, Exp (*B*), of a continuous predictor is the multiplicative effect on the outcome of a one unit change in the predictor when the other continuous predictors are equal to 0 and the categorical predictors are at their reference category. For categorical predictors, the exponentiated model coefficient for category J represents the ratio of the mean of the category J and the mean of the reference category

^b^Inclusion of the cancer group variable in the model indicated that MEs of breast and lung cancer types did not differ significantly from the MEs of subjects in the “other” category. Therefore, those parameters were constrained to zero

^c^When MCS was excluded from the model, Akaike information criteria increased 5% from 10,137.8 to 10,667.9 and Bayesian information criteria (BIC) increased 5% from 10,163.2 to 10,689.4

The parameters of the final model linking SF-6D to MEs are displayed in Table 5. Age (*P* = 0.001), marital status (*P* = 0.010), and health insurance (*P* = 0.004) were significantly associated with MEs. As in the case of the PCS model described previously, parameters for the lung and breast cancer groups were constrained to zero given their MEs did not differ significantly from those of the “other” group. For the SF-6D model, tests for model coefficients representing interactions between cancer groups and SF-6D utility score indicated that the association between the SF-6D and MEs was significantly different for patients with prostate cancer when compared to patients with the “other” cancer category (*P* = 0.003), but not for the remaining cancer groups (*P* > 0.05). SF-6D differences had the strongest impact in the prostate cancer group with a 0.05 point higher (better) SF-6D score associated with approximately 30% (RR = 1.230; 95% CI [1.160, 1.440]) lower MEs. In other cancer groups, a 0.05 point higher SF-6D score was associated with approximately 8% (RR = 1.080; 95% CI [1.025, 1.135]) lower MEs.

**Table 5:** Model parameters for final model linking SF-6D and medical expenditures, after inclusion of cancer types

Parameter	Exp (*B*)^a^	SE (*B*)	*Z*	*P* value	95% CI
Constant	3743.75	2114.89	14.56	0.000	1237.24–11,328.19
SF-6D^c^	**0.21**	**0.11**	**− 2.98**	**0.003**	**0.08–0.59**
Cancer type—other (reference)					
Prostate	5.47	5.31	1.75	0.080	0.82–36.66
Breast	1.00	(restricted)^b^			
Skin	**0.35**	**0.08**	**− 4.62**	**0.000**	**0.23–0.55**
Lung	1.00	(restricted)^b^			
Cancer type by PCS (interaction)					
Prostate	**0.02**	**0.03**	**− 2.94**	**0.003**	**0.00–0.29**
Breast	1.00	(restricted)^b^			
Skin	1.00	(restricted)^b^			
Lung	1.00	(restricted)^b^			
Age	**2.69**	**0.84**	**3.18**	**0.001**	**1.46–4.96**
Gender (male)	1.32	0.23	1.58	0.113	0.94–1.86
Education—< HS (reference)					
HS or higher	1.36	0.23	1.80	0.072	0.97–1.90
Married	**0.67**	**0.10**	**− 2.59**	**0.010**	**0.50–0.91**
Insured	**2.34**	**0.70**	**2.85**	**0.004**	**1.30–4.20**

Values shown in bold are statistically significant (*P* < 0.05) model parameters

*PCS* physical component summary, *SE* standard error, *HS* high school, *CI* confidence interval

^a^For the GLM with log link, the exponentiated coefficient, Exp (*B*), of a continuous predictor is the multiplicative effect on the outcome of a one unit change in the predictor when the other continuous predictors are equal to 0 and the categorical predictors are at their reference category. For categorical predictors the exponentiated model coefficient for category J represents the ratio of the mean of the category J and the mean of the reference category

^b^Inclusion of the cancer group variable in the model indicated that MEs of breast and lung cancer types did not differ significantly from the MEs of subjects in the “other” category. Therefore, those parameters were constrained to zero

^c^When SF-6D was excluded from the model, Akaike information criteria increased 8% from 9795 to 10,580 and the Bayesian information criteria increased 8% from 9837 to 10,614

**Fig. 2:**
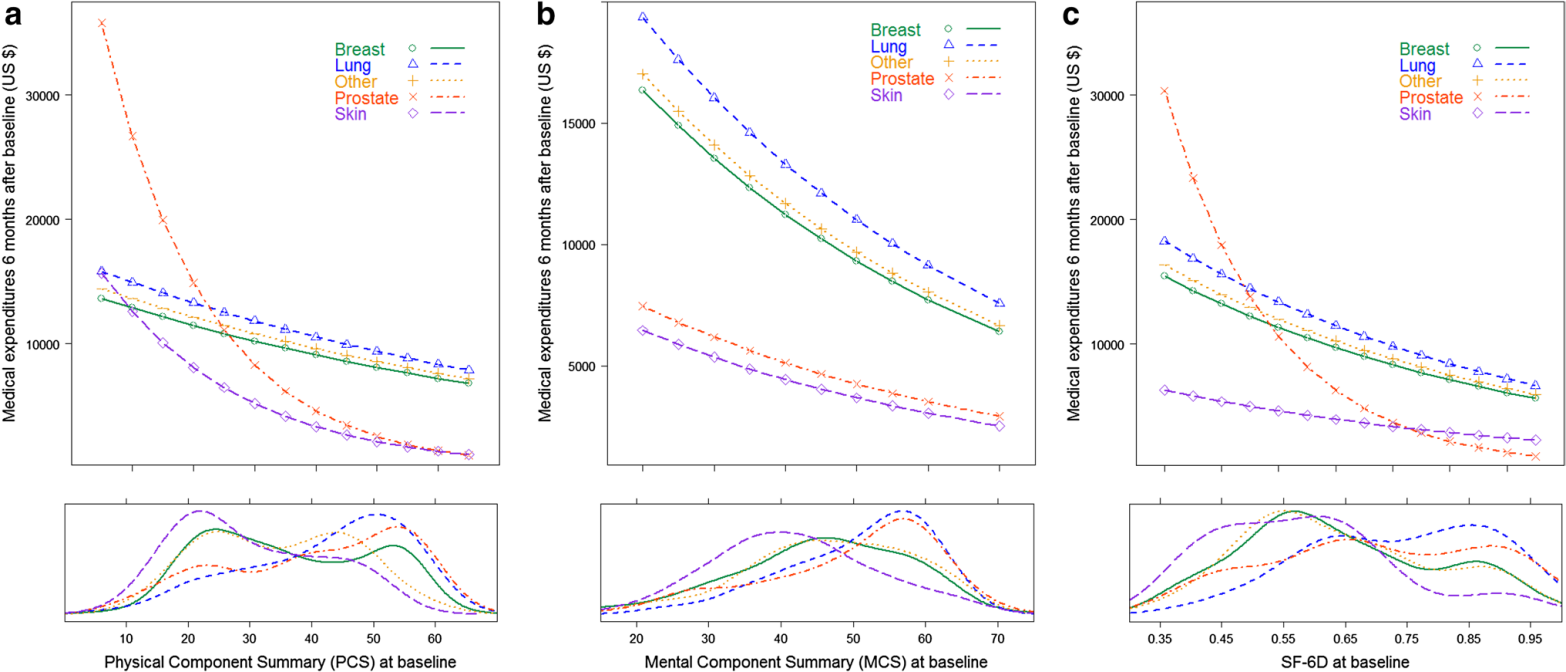
Estimated medical expenditures as a function of HRQoL and utility scores and scores distribution (bottom panels), by cancer type

Figure 2, corresponding to Tables 3, 4, and 5, depicts the association between PCS and MEs (Fig. 2a), MCS and MEs (Fig. 2b), and SF-6D and MEs (Fig. 2c) for the five cancer types, with the lower panels of Fig. 2 showing the smoothed distribution of PCS, MCS, and SF-6D scores over the range of predicted MEs. For PCS, the prostate cancer type has the steepest line (strongest effect of PCS on MEs), followed by skin cancer, while the curves for the remaining three cancer types are parallel on the log scale (indicating 1% lower ME for a 1-point better score across all three cancer groups). Differences in PCS score of the magnitude of the MID of three points established for the SF-12v2 [19] were associated with 3% lower MEs for patients in the “other,” “breast,” and “lung” cancer types, 12% lower MEs for “skin” cancer, and 17% lower MEs for “prostate” cancer. Differences in MCS of the magnitude of the MID (3 points) [19] were associated with 7.5% lower MEs, across all cancer groups. For SF-6D, an MID of 0.041 has been proposed [28]. This score difference was associated with 24.0% (95% CI [1.130, 1.349]) lower MEs for “prostate” cancer and 6.5% (95% CI [1.021, 1.110]) lower MEs for other cancers. The distribution curves in the lower panels of Fig. 2 indicate most patients with skin and prostate cancer were in the higher range of the PCS scale while patients in the lung cancer groups tend to be more concentrated in the lower PCS values (< 30). Differences across cancer groups in terms of MCS and SF-6D scores were similar to those observed for PCS scores, with patients with lung cancer more concentrated towards the lower end of the score range, whereas patients with skin and prostate cancer tended to be more concentrated in the higher values.

### Association between SF-12v2 scores and utilization frequency

**Fig. 3:**
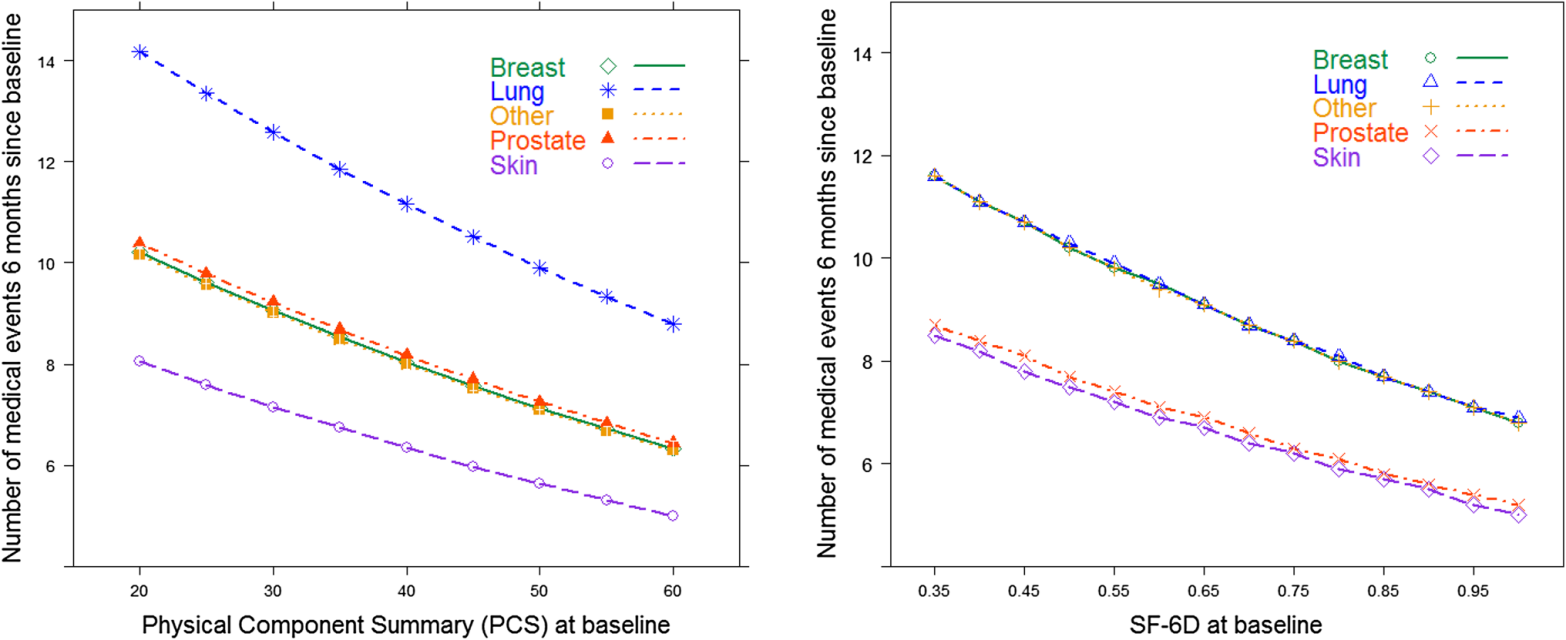
Estimated utilization frequency as a function of physical component summary (PCS) and SF-6D utility score, by cancer type

Table 6 presents the parameters of the final model linking PCS and utilization frequency. The model included PCS, age, gender, education, and cancer type. Utilization frequency for breast and prostate cancer types did not differ significantly from those in the “other” category. Therefore, the parameters for these categories were constrained to zero in the final model. PCS was significantly associated with utilization frequency (*P* = 0.003), with a one-point higher PCS associated with 1% (RR = 1.012; 95% CI [1.004, 1.020]) lower frequency. The interaction between PCS and cancer type did not reveal significant differences in the effect of PCS on utilization across cancer groups (*χ*^2^ = 1.61; *P* = 0.4466). MCS was not found to be significantly associated with utilization frequency (*P* = 0.956). The model for the SF-6D (Table 7) indicated that age (*P* = 0.022) and education (*P* = 0.014) were significantly associated with utilization frequency. As in the case of the PCS model described previously, interaction terms indicated no significant differences in the effect of SF-6D on utilization frequency across cancer groups, and the effect of a 0.05-point higher SF-6D score was estimated to be approximately 4% (RR = 1.041; 95% CI [1.010, 1.073]) lower MEs among all cancer groups (Fig. 3).

**Table 6:** Model parameters for final model linking PCS and utilization frequency, after inclusion of cancer types

Parameter	Exp (*B*)^a^	SE (*B*)	*Z*	*P* value	95% CI
Constant	6.59	2.05	6.06	< **0.001**	3.58–12.14
PCS^c^	**0.99**	**0.00**	**− 3.00**	**0.003**	**0.98–1.00**
Cancer type—“other” (reference)					
Prostate	1.00	(restricted)^b^			
Breast	1.00	(restricted)^b^			
Skin	**0.76**	**0.11**	**− 1.98**	**0.048**	**0.58–1.00**
Lung	**1.43**	**0.25**	**2.08**	**0.038**	**1.02–2.02**
Age	1.35	0.30	1.36	0.175	0.88–2.07
Gender (male)	0.94	0.10	**−** 0.62	0.535	0.76–1.15
Education—< HS (reference)					
HS	**1.57**	**0.23**	**3.11**	**0.002**	**1.18–2.09**
College/assoc. dgr	**2.26**	**0.42**	**4.45**	**0.000**	**1.58–3.25**
College	1.34	0.28	1.39	0.165	0.89–2.01
> College	**1.63**	**0.35**	**2.31**	**0.021**	**1.08–2.47**

Values shown in bold are statistically significant (*P* < 0.05) model parameters

*PCS* physical component summary, *SE* standard error, *CI* confidence interval, *HS* high school

^a^For the GLM with log link, the exponentiated coefficient, Exp (*B*), of a continuous predictor is the multiplicative effect on the outcome of a one unit change in the predictor when the other continuous predictors are equal to 0 and the categorical predictors are at their reference category. For categorical predictors, the exponentiated model coefficient for category J represents the ratio of the mean of the category J and the mean of the reference category

^b^Inclusion of the cancer group variable in the model indicated that MEs of prostate and breast cancer types did not differ significantly from the MEs of subjects in the “other” category. Therefore, those parameters were constrained to zero

^c^When PCS was excluded from the model, Akaike information criteria increased 5% from 4971.05 to 5247.59 and Bayesian information criteria (BIC) increased 5% from 5012.79 to 5285.56

**Table 7:** Model parameters for final model linking SF-6D and utilization frequency, after inclusion of cancer types

Parameter	Exp (*B*)^a^	SE (*B*)	*Z*	*P* value	95% CI
Constant	6.70	2.22	5.73	0.000	3.50–12.84
SF-6D^c^	0.45	0.14	**−** 2.62	0.009	0.24**–**0.82
Cancer type—“other” (reference)					
Prostate	0.67	0.11	**−** 2.44	0.015	0.49–0.92
Breast	1.00	(restricted)^b^			
Skin	0.67	0.08	**−** 3.32	0.001	0.53**–**0.85
Lung	1.00	(restricted)^b^			
Age	1.63	0.35	2.29	0.022	1.07–2.48
Gender (male)	1.05	0.14	0.38	0.707	0.82–1.35
Education—< HS (reference)					
HS	1.46	0.21	2.64	0.008	1.10**–**1.94
> HS	1.53	0.23	2.79	0.005	1.13**–**2.05

*PCS* physical component summary, *SE* standard error, *CI* confidence interval, *HS* high school

^a^For the GLM with log link, the exponentiated coefficient, Exp (*B*), of a continuous predictor is the multiplicative effect on the outcome of a one unit change in the predictor when the other continuous predictors are equal to 0 and the categorical predictors are at their reference category. For categorical predictors, the exponentiated model coefficient for category J represents the ratio of the mean of the category J and the mean of the reference category

^b^Inclusion of the cancer group variable in the model indicated that MEs of prostate and breast cancer types did not differ significantly from the MEs of subjects in the “other” category. Therefore, those parameters were constrained to zero

^c^When SF-6D was excluded from the model, Akaike information criteria increased 6% from 2905.6 to 3091.7 and Bayesian information criteria (BIC) increased 6% from 2938.8 to 3121.2

## Discussion

This study found that PCS, MCS, and SF-6D scores from the SF-12v2 can be used to predict total MEs for patients with cancer. A one-point higher (better) PCS or MCS score was associated with approximately 2% lower MEs, while a 0.05 difference in SF-6D was associated with 9.5% lower MEs. Results also indicated that, for PCS and SF-6D index, the association with MEs varied by cancer type. For patients with prostate and skin cancer, a one-point higher PCS was associated with 6% and 4% lower MEs, respectively. For the remaining cancer groups (“other,” breast, lung), a one-point higher PCS was associated with 1% lower MEs. A 0.05-point higher (better) SF-6D score was associated with approximately 30% lower MEs among patients with prostate cancer, while for other cancer groups, a 0.05-point higher SF-6D score was associated with approximately 8% lower MEs. Additionally, PCS and SF-6D, but not MCS, can also be used to predict utilization frequency. A one-point higher PCS score was associated with a 1% increase in utilization frequency. Similarly, a SF-6D difference of 0.05 was associated with 4% decline in utilization frequency. We found no evidence that either of these associations varied by cancer group. Overall, these results are in agreement with previous studies examining the association between PRO scores and HCRU in patients with cancer [10–14, 16, 29, 30]. Similarly, a recent study found a significant association between health-utility scores (EQ-5D) and social care needs [31].

While the association between PRO scores and HCRU in MEPS cancer survivors has been previously studied [12], the current study established this association specifically in subjects with active disease and used a specific longitudinal link between the time of health status assessment and the period in which MEs were captured, i.e., approximately 6 months following the SF-12v2 administration. Thus, unlike prior studies, these results demonstrated that future HCRU could be predicted by using current HRQoL status and utility scores reported by patients.

While the MCS score was significantly associated with subsequent total MEs, it was not significantly associated with utilization frequency. Another MEPS study found [12], that the effect of psychological distress on total MEs among cancer survivors is largely driven by office-based expenditures and prescription expenditures, the latter of which was not included in this study’s measure of utilization frequency. Similarly, a previous study [10] regarding the associations between various symptoms and emergency room visits found that physical symptoms (appetite, drowsiness, nausea, pain, shortness of breath, tiredness) showed association with emergency visits, while anxiety and depression did not.

While this study represents a promising first step in a sparsely researched area, it is important to be aware of the study limitations. Although the sample size was sufficient to estimate mean expenditures (at 95% confidence level),^1^ group comparisons were limited by sample size. Thus the results from subgroups analyses must be interpreted with caution. Future studies should seek to confirm such relationships in larger samples. In addition, the results may only be relevant to the non-institutionalized population, which is limiting for the target population of the study (patients with cancer not in remission).

In conclusion, this study aimed to improve interpretation of scores based on the SF-12v2, by translating differences in these scores to metrics familiar to medical decision makers, clinicians, and patients alike, such as MEs and utilization frequency. This information is expected to help guide interpretation of PRO data that are increasingly used in medical decision-making and health policy decisions. The policy-related implications of our research suggest that programs that improve the physical functioning, mental functioning, and HRQoL of patients with cancer could have a significant and measurable reduction in their MEs.

## Footnotes

1 Based on prior recommendations [32], given our sample’s skewness for total expenditures (Fisher’s skewness measure *G*1 = 4.42), a sample size ≥ 489 is suitable for estimation of the mean.
